# Creative teaching behaviors of health care school teachers in Taiwan: mediating and moderating effects

**DOI:** 10.1186/s12909-019-1641-8

**Published:** 2019-06-04

**Authors:** Hsing-Yuan Liu, I-Teng Wang

**Affiliations:** 1 School of Nursing, Chang Gung University of Science and Technology, No. 261, Wunhua 1st Rd., Gueishan Township, Taoyuan, Taiwan Republic of China 33303; 2 Associate Research Fellow, Chang Gung Memorial Hospital Taiwan; No. 261, Wunhua 1st Rd., Gueishan Township, Taoyuan, Taiwan Republic of China 33303

**Keywords:** Creative teaching behaviors, Mediated effects, Moderated effects, Health care school teachers

## Abstract

**Background:**

Considerable attention has been paid to the variables for creative abilities of teachers and the creative climate of the schools in which they teach, as well as the direct relationships between these variables. However, research on the indirect links between these variables concerning creativity in higher education has been limited. Researches on creative teaching behaviors among health care teachers are scant, particularly in Taiwan.

**Methods:**

This study used a cross-sectional descriptive design to investigate potential mediating and moderating effects of Taiwanese health care school teachers’ creative teaching self-efficacy and a school’s creative climate, the relationship between these variables, and the relationship between creative teaching self-efficacy and creative teaching behaviors. Participants were purposively selected from five vocational and technical health care schools in Northern Taiwan representing the departments of nursing, gerontological care and management, and nutrition and health. Data were collected from five self-report questionnaires regarding teaching, the school environment, and creativity. Data were analyzed using Pearson’s correlation coefficient and simple and hierarchical multiple regression models.

**Results:**

A total of 53 teachers completed the questionnaires. Pearson’s correlation analysis showed the teamwork component of school creative climate was correlated with the creative teaching behavior of characteristics and motivations. The mediation model indicated creative teaching self-efficacy fully mediated the effect of teamwork on teachers’ characteristics and motivations. The moderation model indicated that teamwork negatively moderated the effect of teachers’ creative teaching abilities for characteristics and motivations on creative teaching behaviors (β = − 0.01, *p* < 0.001).

**Conclusions:**

Our findings fill a gap in the literature regarding creative teaching behaviors and school climate in Taiwan. School teachers’ creative teaching self-efficacy and creative teaching abilities are crucial mediating and moderating variables on the relationship between school creative climate and creative teaching behaviors, respectively. The empirical data confirm the validity of our proposed mediation and moderation models of creative teaching behaviors. Therefore, our findings may be effective references for health care teachers regarding creative teaching. Improving creative teaching behaviors of teachers responsible for educating students in health care schools could be facilitated by enhancing teachers’ creative self-efficacy and creative abilities.

## Background

The Taiwanese government mandated that schools must foster creativity in students at all educational levels (White Paper on Creative Education, Taiwan’s Ministry of Education, 2001). As a result, technical institutes and vocational schools in Taiwan have integrated creativity, innovation, and entrepreneurship into capstone courses since 2006. These courses include healthcare product-based curricula, which numerous health care schools, including nursing programs, have introduced to help students develop creative and innovative abilities in order to solve healthcare problems [1].

Despite the importance of teaching creativity in Taiwanese nursing schools, not every healthcare school teacher is gifted with creativity [2]. Therefore, understanding factors that influence a teacher’s ability to teach creativity could improve student outcomes. Unfortunately, Unfortunately, most research on optimizing teaching creativity in healthcare education has focused on the students [3–7]. Only two recent studies have examined factors influencing the ability of nursing faculty in Taiwan to teach creativity in healthcare education [2, 8]. Because of the rapid changes in global health care, the Taiwanese government has elected to enrich and enhance the ability of teachers to teach creativity; thus, examining factors that influence creative teaching behaviors in health care schools in Taiwan is crucial.

The concept of creativity is not well-defined and is dependent upon the context in which it is being evaluated [8, 9]. Teachers in professional healthcare schools need to teach material creatively in order to help students develop new healthcare products to meet the demands of the growing healthcare market [1]. The measure of creativity can be influenced by social climate or the physical environment of an organization [9]. Therefore, in Taiwan, technology schools often depend on the Creativity Teaching Efficiency of Technology Institute Teacher’s Scale (CTETITS) as a measure of a teacher’s creativity [1]. The CTETITS was designed to specifically address aspects of creative abilities considered to be important for helping technology students successfully develop patentable products.

Several empirical studies have demonstrated a positive relationship between organizational climate and an individuals’ creative and innovative behavior [10–12]. Thus, the creative climate of an academic setting is crucial for promoting creative teaching behaviors [13].

Tierney and Farmer (2002) added the construct of creative self-efficacy as an additional component of creativity [14]. This component measures the level of a person’s belief in their ability to be creative in their work role. Several studies have reported that creative self-efficacy in teaching is positively related to creative teaching behaviors [15–19]. Creative self-efficacy has been shown to have a significant effect on creative behaviors of manufacturing employees [14, 20] and a significant mediating role of creative self-efficacy predicted employees’ creativity [20].

Bandura suggested levels of self-efficacy are also influenced by components of the organizational climate [21]. Several studies have confirmed that a supportive school climate positively influences teachers’ beliefs regarding self-efficacy [22, 23]. Moreover, individuals with high levels of creative self-efficacy exhibit creative behavior when they are in a climate that is supportive of innovation [24]. One can apply these findings to understanding creative teaching: a creative school climate can influence creative teaching behaviors through creative teaching self-efficacy.

A work environment that supports a creative climate plays a crucial role in motivating creativity [24–26]. However, a recent study in Taiwan by Chiu (2017) demonstrated creative teaching behaviors that encouraged diverse viewpoints and adopted diverse evaluations, failed to effectively enhance students’ creativity [27]. Chiu suggested the inability to enhance students’ creativity may have been due to the teacher’s perception that the organizational climate was not innovative, resulting in a reduction in creative teaching self-efficacy, and a negative effect on the effectiveness of creative teaching behaviors [27]. This suggests that creative teaching self-efficacy and a school’s creative climate have a relationship with creative teaching behaviors.

Taken together, these studies suggest a school’s creative climate can influence creative teaching behaviors through creative teaching self-efficacy. The previous findings also suggest there is an effect of the interaction between a school’s creative climate and teaching self-efficacy on creative teaching behaviors. Therefore, this study examined how fostering creativity in Taiwanese healthcare schools might be affected by the potential moderating and mediating roles of the relationship between (1) creative teaching self-efficacy and creative teaching behaviors and (2) a school’s creative climate and creative teaching behaviors. The following two hypotheses guided this research:

- *Hypothesis 1*: Creative teaching self-efficacy mediates the relationship between school creative climate and creative teaching behaviors in healthcare schools.
- *Hypothesis 2*: School creative climate moderates the relationship between creative teaching ability and creative teaching behaviors in healthcare schools.

### Proposed conceptual framework

**Fig. 1:**
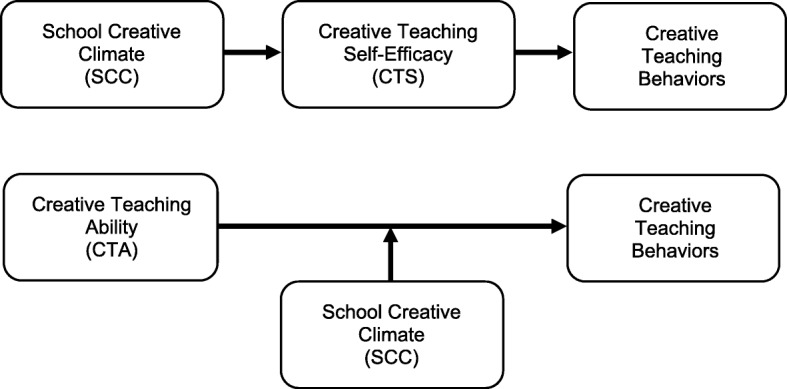
Hypothesized models for mediating (**a**) and moderating (**b**) effects of creative teaching variables

The aforementioned findings on creative teaching self-efficacy informed the proposed conceptual framework for the hypothetical model of our study. We examined two sets of interactions. First, based on Bandura’s theory of self-efficacy [19], we examined whether creative teaching self-efficacy (CTS) would have a mediating effect (partially or fully) on the relationship between school creative climate (SCC) and creative teaching behaviors (CTB) (Fig. 1a). Second, based on Litwin and Stringer’s creative climate model for work environments [28], we hypothesized that school creative climate would have a moderating effect on the association between creative teaching ability and creative teaching behaviors (Fig. 1b). Therefore, teachers with high levels of creative teaching ability in a school with a high level of creative climate should exhibit high levels of creative teaching behaviors. Whereas teachers with high levels of creative teaching ability in schools with low levels of creative climate should exhibit low levels of creative teaching behaviors.

## Methods

### Design

A cross-sectional quantitative design with structured questionnaires was used for this study.

### Participants and setting

Teachers were selected by purposive sampling. An email was sent to four professional healthcare schools in Taiwan asking any teachers interested in participating in the study to respond. A total of 64 teachers expressed interest in participating. We used G*Power to calculate the required minimum sample size and set the confidence level as 95%, the power of test as 0.8, and the number of predictor variables as 3. Subsequently, the minimum sample size required was determined to be 52 [29]. Teachers who expressed interest in participating received a packet containing a description of the study design and purpose, a consent form, and survey questionnaires. Teachers were instructed to sign the enclosed consent form, fill out the questionnaires, and return the packets by mail. Anonymity of the data was maintained by assigning a coding number to each packet. A total of 53 completed packets were returned for a response rate of 82.9%. Participants were employed in the departments of nursing, gerontological care and management, and nutrition and health; the mean age was 49 years (standard deviation [SD] = 4.73); most were female (96%). Approximately 38% of the teachers had at least 19 years of teaching experience. Approximately two-thirds (62%) of the teachers had 3 or more years of creativity teaching experience. In addition, approximately 53% of the teachers had received at least 10 h of creativity training. Slightly over three-quarters (78%) of the teachers expressed an interest in teaching creativity courses.

### Data collection

Data were collected between October 2016 and January 2017 from survey questionnaires. One questionnaire was used to obtain data regarding participants’ demographics (age, gender) and teaching characteristics (teaching experience, creativity training, and interest in teaching creativity courses). Four self-report questionnaires were used to collect data regarding creative teaching abilities (CTA), creative self-efficacy (CSE), creative teaching behaviors (CTB)and school creative climate (SCC). Measurements were determined using the self-report instruments described below.

### Measurements

#### School creative climate

School creative climate was assessed with the School Creative Climate Scale (SCCS), which is an author developed scale modified from the Creativity Working Environment Scale [26]. The SCCS contains statements regarding four qualities of school climate: school encouragement (SE), school support (SS), team cooperation (TC), and sufficient resources (SR). Statements are scored using a 5-point Likert scale from 1 = never to 5 = always. Higher scores indicate greater support for creativity. Factor analysis for this study showed that the communality values for the constructs of the SCCS were between 0.578 and 0.912 and the cumulative variation explained by the SCCS was 82.41%. Cronbach’s alpha coefficient was computed to determine the reliability estimates of the SCCS for this study, which was 0.76. The results indicated that the SCCS had satisfactory validity and reliability. The four statements for the SCCS are shown in Table 1.

**Table 1:** Examples of statements for subscale measures of creativity. The range for all statements is 1–5

Scale	Example statement
SCCS subscales (4)	
School encouragement (SE)	Schools encourage teachers to engage in creative work through public praise or rewards.
School support (SS)	The school supports the creative ideas of the students.
Team cooperation (TC)	Students can exchange ideas with each other without reservation.
Sufficient recourses (SR)	The school provides sufficient supplies for developing healthcare products.
CTBS subscales (4)	
Autonomous learning (AL)	I provide instruction to students on methods to improve learning.
Creative thinking (CT)	I explain to students about obstacles and frustrations that are part of the process of creativity.
Characteristics/motivation (CM) Environment/opportunity (EO)	I provide challenging and exciting materials to my students in accordance with their abilities, to help them master required skills. I encourage students to communicate with other members with positive language in group.
SECTS subscales (3)	
Positive self-appraisal (SA)	I can guide students to use creative thinking strategies to develop their creativity.
Lack of negative consciousness (NC)	I have enough creativity knowledge for teaching work.
Belief in one’s resistance to stress (BRS)	I can actively develop student creativity with my teaching, even if the school atmosphere is not conducive to creativity.
CTETITS subscales (6)	
Ability to evaluate patents and trademarks (EPT)	I encourage students to use their imagination to create trademark designs.
Ability to teach about patents (ATP)	After my teaching, the students’ works are more creative.
Ability to teach creatively (ATC)	I appreciate the students’ creations and provide additional rewards
Knowledge of intellectual property rights (KIPR)	My teaching results in a higher likelihood of my students’ obtaining property rights for the product they created.
Ability to design courses (ADC)	My curriculum design can enhance students’ creativity.
Belief in the creativity of students (BCS)	I think that students’ creativity can be nurtured.

Note: *SCCS* School’s Creative Climate Scale, *CTBS* Creative Teaching Behavior Scale, *CTETIS* Creativity Teaching Efficiency of Technology Institute Teacher’s Scale, *SECTS* Self-efficacy for Creative Teaching Scale

#### Creative teaching behavior (CTB)

The Creative Teaching Behavior Scale (CTBS) measured teachers’ creative teaching behavior. The CTBS is based on the 14-item Creativity Fostering Teacher Behavior Index developed by Soh [23]. Four subscales measure the following teaching behaviors: autonomous learning (AL), creative thinking (CT), characteristics/motivation (CM), and environment/opportunity (EO), which are similar to Amabile’s four components of creativity [24, 25]. AL (four items) determines how teachers foster a student’s independent learning, which involves providing opportunities for exploration, self-teaching, and learning. CT (four items) assesses a teacher’s ability to encourage and explain creative thinking, which includes creative approaches to problem solving as well as obstacles to creativity. CM (four items) determines how teachers encourage students to master basic requirements and approach conflict situations with a positive attitude. EO (two items) is the effort a teacher makes to foster student creativity in small groups through cooperation and interpersonal interactions. Items are scored with a 5-point Likert scale: 1 = never to 5 = always. The subscale score is the mean score for the items; total score is the mean across all four subscales. Cronbach’s alpha for the CTBS was 0.91, and satisfactory validity was established for this scale through factor analysis [11]. In this study the Cronbach’s alpha was 0.89. Table 1 contains example statements for the subscales of the CTBS.

#### Creative teaching self-efficacy (CTS)

We measured creative teaching self-efficacy with the total score for the SECTS, developed by Lin and Chou [9]. The SECTS self-report instrument uses statements to measure three personality traits associated with creative teaching self-efficacy: positive self-appraisal (SA, seven statements), lack of negative consciousness (NC, five statements), and belief in one’s resistance to stress (BRS, three statements). The statements are scored with a 5-point Likert scale; 1 = strongly disagree to 5 = strongly agree. The total score is the average of the sum of the three subscale scores (range = 1–5); a higher score indicates a greater level of creative teaching self-efficacy. The average total score has been reported to be 3.94; the average score for the three subscales is between 3.51 and 4.34 [9]. Cronbach’s alpha coefficient for the SECTS is 0.92. Confirmatory factor analysis (CFA) showed the factor loadings for the statements for SA, NC, and BRS were 0.632–0.905, 0.615–0.835, and 0.769–0.98, respectively. The composite reliability (CR) for the SA, NC, and BRS was 0.906, 0.805, and 0.917, respectively; the average variance extracted (AVE) of the SA, NC, and BRS was 0.58, 0.584, and 0.789, respectively. Therefore, CFA established satisfactory validity for the SECTS instrument. Reliability of the SECTS for this study, determined by Cronbach’s alpha coefficient, was 0.76. Examples of statements used to measure the three traits of the SECTS are shown in Table 1.

#### Creative teaching ability (CTA)

Creative Teaching Ability (CTA) was measured with the total score of the Creative Teaching Efficiency of Technology Institute Teacher’s Scale (CTETITS) [2]. The CTETITS is a 31-item self-report instrument used to assess abilities considered important for teaching technology creatively. Four abilities are measured with the following subscales: evaluate patents and trademarks (EPT; 11 items), teach about patents creatively (TPC;), ability to teach creatively (ATC; 5 items), knowledge of intellectual property rights (KIPR; 7 items), creatively design courses (CDC; 3 items), and ability to believe in the creativity of students (BCS; 2 items). Each item is represented by a statement and scored on a 5-point Likert scale. For example, the EPT item states, “My ability to evaluate patents and trademarks is excellent”; 1 = strongly disagree; 2 = disagree; 3 = neutral; 4 = agree; or 5 = strongly agree. The total score for the CTETITS is the average of the sum of the six abilities; higher scores indicate a greater perceived ability to teach technology creatively. The average total score has been reported to be 3.57; average subscale scores for qualities range from 3.22 to 4.09 [2]. Cronbach’s alpha coefficient for the CTETITS is 0.97. Factor analysis established satisfactory validity for the scale [2]. Reliability of the CTETITS for this study, determined by Cronbach’s alpha coefficient was 0.93. Table 1 shows examples of statements for each subscale.

#### Reliability of the instruments

Table 2 shows the Cronbach’s α coefficients for the four scales and subscales used in this study. The SCCS, CTBS, SECTS, and CTETITS measure school creative climate, creative teaching behaviors, creative teaching self-efficacy, and creative teaching ability, respectively. All Cronbach’s α coefficients are greater than 0.7, indicating the scales and subscales have satisfactory reliability.

**Table 2:** Cronbach’s alpha coefficients of the scales and subscale of the instruments

Instrument	Cronbach’s α-coefficient
School Creative Climate Scale (SCCS)	
Total scale (15 items)	0.76
Subscales	
School encouragement (SE; 4 items)	0.89
School support (SS; 5 items)	0.76
Teamwork (TW; 3 items)	0.91
Sufficient resources (SR; 3 items)	0.93
Creative Teaching Behavior Scale (CTBS)	
Total scale (14 items)	0.81
Subscales	
Autonomous learning (AL; 4 items)	0.83
Creative thinking (CT; 4 items)	0.77
Characteristics and motivations (CM; 4 items)	0.87
Environment and opportunity (EO; 2 items)	0.70
Self-Efficacy for Creativity Teaching Scale (SECTS)	
Total (15 items)	0.81
Subscales	
Self-affirmation (SA; 7 items)	0.90
Negative consciousness (NC; 4 items)	0.73
Stress resistant beliefs (SRB; 4 items)	0.75
Creativity Teaching Efficiency of Technology Institute Teachers Scale (CTETITS)	
Total scale (31 items)	0.93
Subscales	
Evaluate patents and trademarks creatively (EPTC;11 items)	0.92
Teach patents creatively (TPC; 3 items)	0.83
Ability to teach creatively (ATC; 5 items)	0.83
Knowledge of intellectual property rights (KIPR; 7 items)	0.77
Creatively design courses (CDC; 3 items)	0.86
Believe in the creativity of students (BSC; 2 items)	0.91

#### Data analysis

After all packets were collected, data were entered into a computer and analyzed using SPSS version 20.0. Descriptive statistics using the mean and standard deviation (SD) evaluated the characteristic of the participants. Analysis with Pearson’s correlation coefficient identified correlations between school creative climate, creative teaching behaviors, creative teaching ability and creative teaching self-efficacy. Subsequently, stepwise linear regression and hierarchical multiple regression were performed to test the mediating and moderating effects of creative teaching ability and creative teaching self-efficacy on school creative climate and creative teaching behavior.

## Results

### Mean scale scores and correlation for SCCS, CTBS, SECTS, and CTETIS

Mean scale scores for the 53 teachers in our study are shown in Table 3. The total score for the CTBS was highest (mean = 4.35, SD = 0.47), and autonomous learning was the highest subscale score (4.56, SD = 0.47) suggesting a high level of creative teaching behaviors for teachers. The SCCS had the lowest mean score (mean = 3.61, SD = 0.70) as well as a low subscale score for sufficient resources (mean = 2.78, SD = 1.15). Mean scores for creative teaching self-efficacy (SECTS) and creative teaching ability (CTETIS) were similar (mean = 3.76, SD = 0.50 and mean 3.95, SD = 0.48, respectively).

**Table 3:** Mean scale and subscale scores for teachers (*N* = 53)

Scale	Mean score	SD
SCCS		
Total	3.61	0.70
Subscale		
SE	3.79	0.90
SS	3.75	0.85
TW	3.97	0.66
SR	2.78	1.15
CTBS		
Total	4.35	0.47
Subscale		
AL	4.56	0.47
CT	4.43	0.52
CM	4.11	0.67
EC	4.25	0.70
SECTS (total)	3.76	0.50
CTETITS (total)	3.93	0.48

Note: *SD* Standard deviation

Analysis of Pearson’s correlations was conducted to determine if there were any relationships between measures for teachers’ school creative climate, as measured with subscales of the SCCS, creative teaching behaviors, measured with subscales of the CTBS, total SECTS scores as measures of creative teaching self-efficacy, and total score for the CTETIS, as a measure of creative teaching ability (Table 4). Correlations were determined using the method of Cohen (1992) [29]. The teamwork (TW) subscale of the SCCS was moderately correlated with the CTBS subscale of characteristics and motivations (CM) (r = 0.35, *p* < .01), suggesting a relationship between these aspects of creative school climate and creative teaching behaviors, respectively. Teachers’ creative teaching ability (CTA) was correlated with creative teaching behavior; the total score for the CTA and all four subscales of the CTBS was moderate (EO, r = .32; *p* < .05) to high (Al, r = .58; CT, r = .60; CM, r = 0.78; p < .01). The total score for the SECTS was significantly correlated with three subscales of the CTBS (p < .01): AL, r = .33; CT, r = .36; and CM, r = .63, suggesting a relationship between creative teaching self-efficacy and creative teaching behaviors, with the exception of environment and opportunity.

**Table 4:** Correlations among participants’ subscale scores of the School Creative Climate Scale (SCCS) and Creative Teaching Behavior Scale (CTBS), and total scores for Self-efficacy for Creative Teaching Scale (SECTS) and Creative Teaching Efficiency of Technology Institute Teacher’s Scale (CTETITS)

Instrument/Subscale	SCCS				CTBS				SECTS	CTETITS
	1	2	3	4	5	6	7	8	9	10
SCCS										
1. School encouragement (SE)	–									
2. School support (SS)	.73**	–								
3. Teamwork (TW)	.33*	.31*	–							
4. Sufficient resources (SR)	.45**	.56**	.20	–						
CTBS										
5. Autonomous learning (AL)	.15	.18	.05	.18	–					
6. Creative thinking (CT)	−.01	.04	.08	.18	.72**	–				
7. Characteristics and motivation (CM)	.22	.19	.35**	.07	.64**	.64**	–			
8. Environment and opportunity (EO)	.05	.27	.06	.26	.43**	.46**	.27	–		
9. SECTS	.13	.24	.34*	.20	.33*	.36**	.63**	.05	–	
10. CTETITS	.04	.01	.24	−.03	.58**	.60**	.78**	.32*	.53**	–

Note: Total score for SECTS measures creative teaching self-efficacy; total score for the CTETITS measures creative teaching ability. * *p* < .05, ** *p* < .01

### Mediation analysis for teachers’ creativity teaching self-efficacy on school creative climate and creative teaching behavior

Analysis with Pearson’s correlation coefficients, shown in Table 4, indicated only the SCCS subscale of teamwork, was correlated with the CTBS subscale of characteristics and motivations. This suggested a potential mediator existed for these two variables. Therefore, we examined whether the relationship of a school creative climate of teamwork (TW) and the creative teaching behavior of characteristics and motivations (CM) were mediated by a teacher’s creative teaching self-efficacy. Following the guidelines suggested by Baron and Kenny [30] an analysis of mediation was performed. Prior to conducting the mediation analysis, we examined the variables for multicollinearity. As shown in Table 4, no correlation coefficient exceeded 0.8, indicating there was no multicollinearity [31].

We used the multiple regression approach recommended by Baron and Kenny [30] to determine if creative teaching self-efficacy mediated TW and CM. Table 5 summarizes the parameters for the multiple regression models. In Model 1, we regressed teachers’ characteristics and motivations on teamwork; the coefficient for teamwork was positive and significant (β = 0.35, *p* < .05). Next, in Model 2 we regressed teachers’ creative teaching self-efficacy on teamwork; and found that it was positive and significant (β = 0.34, p < .05). To meet the third requirement for mediation, we regressed characteristics and motives on teamwork and creative teaching self-efficacy (Model 3). Teachers’ creative teaching self-efficacy was positively and significantly related to teachers’ characteristics and motives (β = 0.57, *p* < .001); teamwork was positive but not significant (β = 0.16, *p* = 0.18). Therefore, our results indicated that teachers’ creative teaching self-efficacy fully mediated the effect of teamwork on teachers’ characteristics and motivations.

**Table 5:** Parameters for the regression analysis of creative teaching self-efficacy (CTS) as a mediator for teamwork (TW) and characteristics and motivations (CM)

Parameter	CTS (mediator)	CM (criterion)	△F	Adjusted R^2^	△R^2^
Model 1; predictor = TW		0.35*	7.23**	0.11	0.12
Model 2; predictor = TW	0.34*		6.67*	0.10	0.12
Model 3; predictor = TW		0.16	17.62***	0.39	0.41
Model 3; mediator = CTS		0.57***			

Note: * *p* < .05, ** *p* < .01, *** *p* < .001

#### Moderation analysis for school creative climate on teachers’ creative teaching ability and creative teaching behavior

**Fig. 2:**
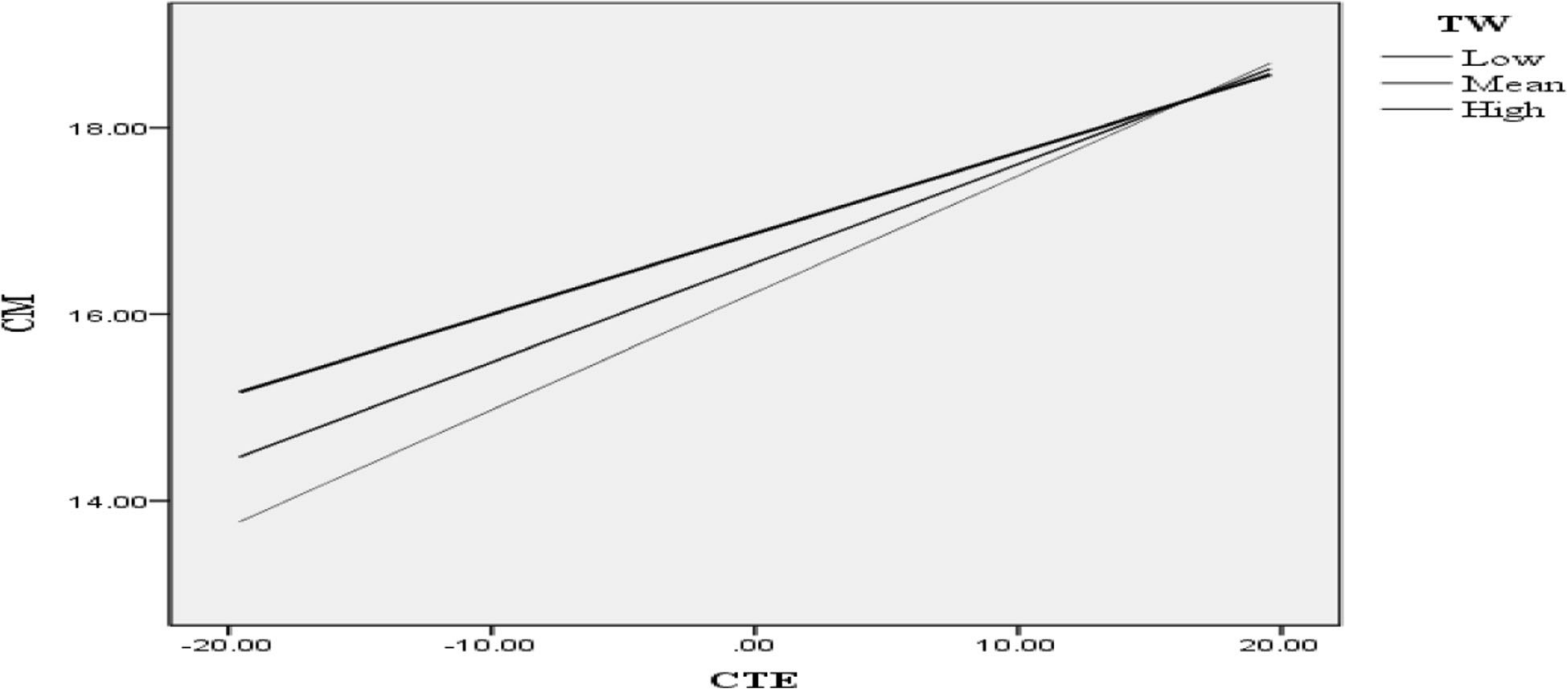
Graph for moderation regression analysis

Before conducting the regression analysis for moderating effects, we used mean centering (subtracting raw scores from the mean) to avoid multicollinearity. To determine whether a moderated relationship existed, we regressed teachers’ characteristics and motivations on teamwork, teachers’ creative teaching ability, and its interaction with teamwork (TW x CTA) using the PROCESS macro available for SPSS and SAS [32]. Table 6 and Fig. 2 show the results of our regression analysis. Table 6 shows the coefficient β was insignificant for teamwork (β = 0.13, *p* = 0.16). However, the coefficient was significant for creative teaching ability (β = 0.11, *p* < .001); there was a negative interaction between teamwork and creative teaching ability (TW x CTA) (β = − 0.01, *p* < 0.001).

**Table 6:** Parameters for moderated regression analysis of teamwork (TW) and creative teaching ability (CTA)

Variable	β	s.e. (β)	t	*p*-value
Constant	16.55 [16.11, 16.98]	0.22	76.65	p < 0.001
TW (centered)	0.13 [0.05, 0.32]	0.09	1.43	p = 0.16
CTA (centered)	0.11 [0.09, 0.12]	0.01	14.31	p < .001
TW x CTA	−0.01 [−0.01, 0.00]	0.01	−3.87	p < .001

The graph in Fig. 2 shows the intersection between creative teaching abilities for low, medium, and high levels of teamwork. These results confirm teamwork had a moderating effect on the relationship between creative teaching abilities and characteristics and motivations.

## Discussion

This study examined the mediating and moderating effects of Taiwanese health care school teachers’ creative teaching ability and teaching self-efficacy on the association between school creative climate and creative teaching behaviors. Our findings indicated that creative school climate is a crucial component for creative teaching behavior, which is in agreement with a prior study by Hsu et al. [13]. In addition, our findings revealed that teachers’ creative self-efficacy can predict creative teaching behaviors; this result was in agreement previous reports [16, 17].

When we examined models for mediating effects, health care school teachers’ creative teaching self-efficacy fully mediated the predictive association between school creative climate and creative teaching behaviors. Specifically, the association between teamwork and characteristics and motivations was fully mediated by creative teaching self-efficacy. Therefore, *Hypothesis 1* was confirmed; the relationship between school creative climate and creative teaching behaviors in healthcare schools is mediated by creative teaching self-efficacy. Our findings suggest teamwork plays an important role in school creative climate, by influencing teachers’ creative teaching behaviors of characteristics and motivations. Therefore, health care schools should encourage a culture of teamwork and mutual support as part of the school’s creative climate in order to enhance creative teaching behaviors, which could improve health care students’ creativity.

The school creative climate variable of teamwork had a moderate (β = 0.34) direct effect on teachers’ creative teaching self-efficacy; teachers’ creative teaching self-efficacy had a large (β = 0.57) direct effect on the creative teaching behavior variable of characteristics and motivations. The indirect (mediated) effect of teamwork on teachers’ characteristics and motivations was small (β = 0.16). Moreover, characteristics and motivations explained 10% of the variance for teamwork (adjusted R^2^ = 0.10). When this was added to creative teaching self-efficacy, characteristics and motivations increased the variance to 39% (adjusted R^2^ = 0.39). This result suggests that creative teaching self-efficacy mediates the association between characteristics and motivations and teamwork among health care teachers. These findings suggest that interventions targeted at improving teachers’ levels of creative teaching self-efficacy may help health care schools with low levels of teamwork stimulate teachers’ creative teaching behaviors involving characteristics and motivations.

When we examined models for moderating effects, we found school creative climate moderated the relationship between creative teaching ability and creative teaching behavior. These findings confirm *Hypothesis 2*: the relationship between creative teaching ability and creative teaching behaviors for health care school teachers is moderated by creative school climate. This result is in agreement with the findings of Hsu et al. (2011); creative teaching ability and school creative climate interacted in a manner to influence creative teaching behaviors among elementary, junior high, and senior high school teachers in Taiwan [13]. Interestingly, Hsu et al. identified only one of eight subscales for school creative climate (team operation) as a moderator. However, our findings are in contrast to those of Cayirdag (2017) who found no association between creative teaching behaviors and creative teaching ability [17].

Teamwork had a positive moderating effect on the interaction of teachers’ creative teaching ability and the creative teaching behavior of characteristics and motivations. This finding suggests teachers with lower levels of creative teaching ability in a school climate with a high level of teamwork should demonstrate stronger creative teaching behaviors. Teamwork had a negative moderating effect on the interaction of creative teaching abilities and creative teaching behaviors involving characteristics and motivations. This suggests teachers with lower creative teaching abilities would demonstrate lower creativity teaching behaviors when they perceive the school climate does not encourage teamwork.

Our study findings fill a gap in the literature regarding the interactions between a school’s environment and how a teacher’s creativity might be fostered. A school’s creative climate and creative teaching behaviors of the teaching staff could be the focus of developing strategies for improving creative teaching self-efficacy. Our findings also suggest that teachers with high levels of creativity teaching ability are more likely to exhibit high levels of creative teaching behaviors.

### Practical implications

The results presented here have implications for health care school teachers in Taiwan. The mediating and moderating roles of creative teaching self-efficacy and teaching abilities should be considered when examining how to improve a school’s creative climate and teachers’ creative teaching behaviors. The mediation and moderation models of creative teaching behavior could be effective references for improving health care teachers’ creative teaching. Enhancing teachers’ creativity, teaching self-efficacy and teaching abilities may be crucial for implementing creativity in health care schools.

The capstone courses for innovative and patentable healthcare products are designed to help nurses become competitors in the international market and solve real-world problems in healthcare (Ku et al., 2014) [5, 6]. Therefore, it is crucial for teaching faculty in nursing programs have knowledge about developing innovative products and applying for patents. Thus the use of the CTETITS was important in this study. One recent study by Liu et al. [1] showed low subscale scores on the CTETITS might be related to the number of hours of teacher training. The association between personality traits, as measured with the SECTS instrument and self-efficacy of teaching intellectual property rights, as measured with the CTETITS, should be further investigated by assessing student creativity as an outcome measure.

#### Limitations

This study had several limitations. First, the sample used was based on a cross-sectional descriptive study design with 53 health care school teachers in Taiwan, which limits the generalizability of our findings to health care teachers outside of Taiwan. However, our data were obtained from five health care schools in Taiwan offering capstone courses related to creativity, which provides a broad perspective within this population. Second, because creative teaching self-efficacy is likely to be a component of a multitude of mediational pathways involved in the association between school creative climate and creative teaching behavior, testing the role of other attributes as explanatory mechanisms is vital for future research. Third, it is a difficult task to compare our results with those of previous studies due to the focus of teaching for creativity of healthcare education is noticeably absent from the literature.

## Conclusions

In this study, we explored the mediating and moderating effects of the relationship between school creative climate and creative teaching behaviors as well as the relationship between creative teaching ability and creative teaching behaviors that foster creativity for teachers in health care schools in Taiwan. Our study confirmed that health care school teachers’ creative self-efficacy and school creative climate are crucial mediating and moderating variables on the relationship between school creative climate and creative teaching behaviors and between teachers’ creative teaching ability and creative teaching behaviors that foster creativity, respectively. The proposed mediation and moderation models of creative teaching behavior were validated by the empirical data and can be used as references for health care teachers’ creative teaching.

This study examined aspects of creative teaching behaviors from a quantitative perspective in or orders to establish a means of evaluating changes in behaviors that can be replicated in other teaching environments. To implement creative education in health care schools, enhancing teachers’ creative teaching self-efficacy and developing creative school climates that encourage teamwork should be key considerations. The measurement scales here could be used to assess the success of such changes. However, additional information on how to implement creative education in health care schools could be gained by conducting qualitative studies to determine teachers’ personal attitudes about creativity and how teamwork might be encouraged.

## Data Availability

The datasets used and/or analyzed during the present study available from the corresponding author on reasonable request.

## Abbreviations

CTB: Creative teaching behaviors
CTETITS: Creativity Teaching Efficiency of Technology Institute Teacher’s Scale
CTS: Creative teaching self-efficacy
SCC: School creative climate
